# Diabetische Neuropathie und diabetischer Fuß (Update 2026)

**DOI:** 10.1007/s00508-025-02675-8

**Published:** 2026-04-30

**Authors:** Gerd Köhler, Astrid Feder, Daniel Hochfellner, Marlies Eichner

**Affiliations:** 1 Klinische Abteilung für Endokrinologie und Diabetologie, Medizinische Universität Graz, Graz, Österreich; 2 Rehabilitationszentrum Aflenz für Stoffwechselerkrankungen mit Schwerpunkt Diabetes mellitus und Adipositas, Aflenz, Österreich; 3 1. Medizinische Abteilung mit Diabetologie, Endokrinologie und Nephrologie, Klinik Landstraße, Wien, Österreich; 4 3. Medizinische Abteilung für Stoffwechselerkrankungen und Nephrologie, Klinik Hietzing, Wien, Österreich

**Keywords:** Diabetische Neuropathie, Therapie schmerzhafte Neuropathie, Diabetischer Fuß, Charcot-Fuß, Diabetic neuropathy, Treatment of painful neuropathy, Diabetic foot, Charcot foot

## Abstract

Der Begriff der diabetischen Neuropathie ist eine Sammelbezeichnung für Erkrankungen des peripheren Nervensystems, die als Spätkomplikation des Diabetes mellitus auftreten. Die Leitlinienempfehlungen beschreiben die klinischen Symptome und diagnostischen Möglichkeiten sowie die Therapiemaßnahmen insbesondere bei der schmerzhaften Form der sensomotorischen Neuropathie einschließlich der komplexen Problematik des diabetischen Fußes.

## Abstract (en)

The term diabetic neuropathy is a collective description for diseases of the peripheral nervous system, which occur as late complications of diabetes mellitus. The guideline recommendations describe the clinical symptoms and diagnostic options as well as the treatment measures, particularly for the painful form of sensorimotor neuropathy, including the complex problem of the diabetic foot.

Änderungen unserer Leitlinie „Diabetische Neuropathie und diabetischer Fuß (Update 2026)“ umfassen die Aktualisierung der aktuellen Evidenz mit Verbesserung der Übersichtlichkeit.

## Diabetische Neuropathie

Unter dem Begriff diabetische Neuropathie werden verschiedene Erkrankungen des peripheren sensomotorischen und autonomen Nervensystems zusammengefasst, die infolge des Diabetes mellitus ohne andere Ursache auftreten. Aufgrund spärlicher Datenlage wird die Prävalenz der diabetischen Neuropathie mit einer großen Varianz angegeben – bei Menschen mit Diabetes mellitus Typ I von 8–54 %, mit Diabetes mellitus Typ II von 13–46 % [1]. Anzeichen einer diabetischen Neuropathie können bereits bei Menschen mit beeinträchtigter Glukosetoleranz bestehen. In der MONICA/KORA-Studie betrug die Prävalenz der Neuropathie rund 30 % bei manifestem Diabetes und 13 % bei Menschen mit gestörter Glukosetoleranz [2].

Risikofaktoren für die Entwicklung einer diabetischen Neuropathie sind Alter, Diabetesdauer, Diabeteseinstellung (Hyperglykämie), Übergewicht und Adipositas. Weitere Risikofaktoren sind arterielle Hypertonie, Bewegungsmangel, Dyslipidämie, Nikotin- und Alkoholkonsum. Auch Komorbiditäten wie die diabetische Retinopathie und die diabetische Nephropathie sowie eine periphere arterielle Verschlusskrankheit (pAVK) erhöhen das Risiko für das Auftreten einer diabetischen Neuropathie [3].

Diabetische Neuropathien können nach Thomas und Tomlinson in symmetrische Neuropathien, fokale und multifokale Neuropathien und Mischformen eingeteilt werden (Tab. 1; [4]).

**Tab. 1:** Einteilung diabetische Neuropathien

Symmetrische Neuropathien	– Sensible oder sensomotorische Polyneuropathie – Autonome Neuropathie – Symmetrische proximale Neuropathie der unteren Extremitäten
Fokale und multifokale Neuropathien	– Kraniale Neuropathie – Mononeuropathie des Stammes (diabetische Radikulopathie) und der Extremitäten – Asymmetrische proximale Neuropathie der unteren Extremitäten (diabetische Amyotrophie)
Mischformen	

## Klinisches Erscheinungsbild der diabetischen Neuropathie

Die diabetische Neuropathie hat verschiedene klinische Erscheinungsbilder. Hierzu gehören die distale symmetrische sensomotorische Neuropathie, die Mononeuropathie, die diabetische Radikulopathie, die diabetische Amyotrophie und die autonome Polyneuropathie.

### Distale symmetrische sensomotorische Neuropathie

Die distale symmetrische sensible Neuropathie stellt mit bis zu 70 % die klinisch häufigste Manifestationsform dar [5]. Typische Symptome sind Taubheitsgefühl, Parästhesien und/oder Schmerzen an den unteren und oberen Extremitäten. Die Beschwerden breiten sich strumpf- bzw. handschuhförmig von distal nach proximal aus, die Schmerzcharakteristik wird als brennend, bohrend und krampfartig beschrieben („burning feet“), und häufig zeigt sich eine Zunahme während der Nachtstunden.

Klinisch finden sich abgeschwächte oder fehlende Eigenreflexe, Sensibilitätsstörungen, ein herabgesetztes Vibrationsempfinden (Pallhypästhesie) und ein gestörtes Temperaturempfinden (Thermhypästhesie). Ausgeprägte Tiefensensibilitätsstörungen können zu einer sensorischen Ataxie führen. Verzögerungen der Nervenleitgeschwindigkeit und eine Amplitudenreduktion der Nervenaktionspotenziale werden durch elektrophysiologische Untersuchungen erfasst.

Die schmerzhafte diabetische Neuropathie beruht vorwiegend auf Veränderungen der schmerzleitenden dünn-myelinisierten Nervenfasern. Die seltene akute schmerzhafte Neuropathie kann bei Therapieintensivierung eines schlecht eingestellten Diabetes auftreten.

Bei einem zusätzlichen Befall der motorischen Fasern finden sich von distal nach proximal fortschreitende Paresen. Die Atrophie der Fußmuskeln führt zu typischen Fußformveränderungen, wie z. B. Krallenzehen, prominentem Mittelfußbereich und einem hohen Längsgewölbe. Die Ausprägung ist sehr unterschiedlich.

Für das Screening auf eine distale symmetrische sensible Neuropathie werden folgende Punkte empfohlen [1]:

- Anamnese (persönliche Basisdaten, diabetesspezifische Daten, Risikofaktoren, Komorbiditäten, bisheriger Verlauf, vorausgegangene Läsionen),
- Erfassung neuropathischer Plus- und Minussymptome (ggf. mithilfe validierter Fragebögen),
- Inspektion und klinische Untersuchung der Beine und Füße,
- Untersuchung auf eine PAVK (Pulsstatus, ggf. Knöchel-Arm-Index [ABI]),
- neurologische Untersuchungen (ggf. mithilfe validierter Scores):
  - Vibrationsempfindung mit C128/64-Hz-Stimmgabel (nach Rydel-Seiffer) oder Druck- bzw. Berührungsempfindung mit dem 10 g-Monofilament und
  - Schmerzempfindung, z. B. mittels 512-mN-Pinprick-Stimulatoren (oder Ähnlichem), oder Temperaturempfindung, z. B. mit stiftförmigem Instrument mit flachem Kunststoff- und Metallende,
  - ggf. zusätzlich Prüfung der Achillessehnenreflexe.

Alle Menschen mit Diabetes müssen regelmäßig auf das Vorliegen einer diabetischen sensomotorischen Neuropathie gescreent werden. Die Erstuntersuchung dazu sollte bei Menschen mit Typ-2-Diabetes zum Zeitpunkt der Diagnosestellung und bei Menschen mit Typ-1-Diabetes nach einer Diabetesdauer von 5 Jahren erfolgen. Regelmäßige Kontrollen sind in jährlichen Intervallen empfohlen. Bei eingeschränkten Zeitressourcen sollte zumindest eine regelmäßige Inspektion des Fußes erfolgen, und das Druck- und Berührungsempfinden sollten mittels Monofilament getestet werden.

Die klinischen Manifestationsformen der sensomotorischen diabetischen Polyneuropathie können in eine subklinische, chronisch schmerzhafte, akut schmerzhafte und schmerzlose Neuropathie eingeteilt werden (Tab. 2; [1]).

**Tab. 2:** Manifestationsformen der sensomotorischen diabetischen Polyneuropathie

Subklinische Neuropathie	– Keine Beschwerden oder klinische Befunde – Quantitative neurophysiologische Tests (Vibrometrie, quantitative Thermästhesie, Elektroneurographie) sind pathologisch
Chronisch schmerzhafte Neuropathie	– Schmerzhafte Symptomatik in Ruhe (symmetrisch und nachts zunehmend): Brennen, einschießende oder stechende Schmerzen, Parästhesien, Dysästhesien, Taubheitsgefühl, unangenehmes Kribbeln, Schlafstörungen – Sensibilitätsverlust unterschiedlicher Qualität, beidseits reduzierte Muskeleigenreflexe
Akut schmerzhafte Neuropathie	– Symmetrische Schmerzen an den unteren Extremitäten und eventuell auch im Stammbereich stehen im Vordergrund – Eventuell zusätzlich Hyperästhesie – Sensibilitätsstörungen an den unteren Extremitäten oder normaler neurologischer Untersuchungsbefund – Kann mit dem Beginn bzw. einer Intensivierung einer Insulintherapie assoziiert sein („Insulinneuritis“)
Schmerzlose Neuropathie	– Fehlende Symptome bzw. Taubheitsgefühl und/oder Parästhesien – Reduzierte oder fehlende Sensibilität, fehlende Muskeleigenreflexe (insbesondere Achillessehnenreflex), Gangunsicherheit, unbemerkte Verletzungen bzw. Ulzera
Langzeitkomplikationen der distal-symmetrischen Polyneuropathie mit unterschiedlichem Penetrationsgrad	– Neuropathische Fußläsionen, z. B. Fußulkus – Diabetische Neuroosteoarthropathie (DNOAP bzw. Charcot-Arthropathie) – Nichttraumatische Amputation

Differenzialdiagnostisch kommen auch andere Ursachen wie Alkoholabusus, Niereninsuffizienz, Vitamin‑B_12_-Mangel, Hypothyreose, entzündliche Prozesse, Paraproteinämie, Intoxikationen mit Metallen und Toxinen (z. B. Blei, Quecksilber) und Medikamente (wie Nitrofurantoin, Linezolid, Amiodaron oder auch Chemotherapeutika wie Cisplatin) in Betracht. Diese müssen mittels Laborkontrollen und genauer Anamneseerhebung ausgeschlossen werden.

Bei folgenden Befunden sollte eine weitere neurologische Abklärung veranlasst werden [2]:

- ausgeprägte Asymmetrie der neurologischen Ausfälle,
- vorwiegend motorische Ausfälle, Mononeuropathie, Hirnnervenstörung ohne gleichzeitige DSPN,
- rasche Entwicklung bzw. Progression der neuropathischen Störungen,
- Progression der Neuropathie trotz optimierter Diabeteseinstellung,
- Beginn der Symptomatik an den oberen Extremitäten,
- Familienanamnese einer Neuropathie,
- Diagnose durch klinische Untersuchung nicht gesichert,
- keine anderen mikro- oder makroangiopathischen Komplikationen.

### Neurophysiologische Diagnostik

Die neurophysiologische Untersuchung stellt den Goldstandard in der Diagnostik einer diabetischen Neuropathie dar [6]. Die elektrophysiologische Abklärung ermöglicht dabei die Darstellung unterschiedlicher Subformen der diabetischen Neuropathie. Eine gemischte sensomotorische und autonome Neuropathie findet sich bei rund 70 % der Menschen mit Diabetes, eine sensible Neuropathieform bei rund 30 %. Die sensible Neuropathie wird in 3 Subgruppen unterteilt, wobei 2a die dick-myelinisierten Fasern betrifft, 2b die dünn-myelinisierten Fasern und 2c gemischte Fasern. Eine rein motorische oder rein autonome Neuropathie findet sich nur in jeweils < 1 % der Fälle.

Die früheste elektroneurographische Veränderung ist eine Amplitudenabnahme des sensorischen Aktionspotenzials des N. suralis (< 6 μV). Weitere sensitive Parameter für eine diabetische Neuropathie sind die sensible Nervenleitgeschwindigkeit des N. suralis und die motorische Nervenleitgeschwindigkeit des N. peroneus. Die eher milde Verlangsamung der Nervenleitgeschwindigkeit und die Amplitudenabnahme der sensiblen und motorischen Potenziale ist auf einen Axonverlust zurückzuführen. Leitungsblöcke sind an sich untypisch mit Ausnahme von Nervenkompressionssyndromen. Vermehrte temporale Dispersion kommt typischerweise an Engpässen und bei fortgeschrittener Neuropathie vor. An den oberen Extremitäten empfiehlt sich die Untersuchung des N. radialis, der nicht durch Engpässe im Karpaltunnel bzw. Sulcus n. ulnaris beeinträchtigt ist.

Diabetische lumbosakrale Plexopathie bzw. thorakale Radikulopathie sind elektromyographisch durch fokale Denervierung im entsprechenden Myotom gekennzeichnet.

### Mononeuropathie

Sowohl Hirnnerven wie auch periphere Nerven können im Rahmen einer Mononeuropathie betroffen sein mit einem variablen Ausmaß von leichter bis kompletter Parese betroffener Muskeln. Die vorwiegend bei älteren Menschen mit Diabetes zu beobachtende diabetische Ophthalmologie beruht auf Ausfällen im Bereich des 3., 4. und 6. Hirnnerven, führt zu Doppelbildern und orbitalen Schmerzen. Diese Form der Neuropathie zeigt jedoch eine günstige Prognose mit Reversibilität innerhalb von 4 bis 6 Wochen. Periphere Ausfälle werden im Bereich des N. medianus und N. peroneus beobachtet. Auch das Risiko der Entwicklung eines Kompressionssyndroms, insbesondere eines Karpaltunnelsyndroms, ist bei Menschen mit Diabetes erhöht.

### Diabetische Radikulopathie

Diese Form der Neuropathie betrifft die segmentalen thorakalen Spinalnerven. Klinisch finden sich ein- oder doppelseitige gürtelförmige Schmerzen thorakal oder abdominal, Paresen im Bereich der Abdominalmuskulatur und Sensibilitätsausfälle.

### Diabetische Amyotrophie

Die diabetische Amyotrophie ist eine eher seltene Form der diabetischen Neuropathie und tritt vor allem bei Diabetes mellitus Typ 2 und im fortgeschrittenen Lebensalter auf. Im Rahmen einer unilateralen schmerzhaften Neuropathie kann dabei sowohl der lumbosakrale Bereich wie auch der Plexus brachialis betroffen sein. Die Menschen berichten über Schmerzen und deutliche Funktionseinschränkungen. Typisch sind Probleme beim Aufstehen aus dem Sitzen aufgrund rasch progredienter atrophierender Paresen der Oberschenkelmuskulatur. Differenzialdiagnostisch muss diese Form deshalb von orthopädischen Erkrankungen abgegrenzt werden. Die Prognose der diabetischen Amyotrophie ist günstig.

### Autonome Polyneuropathie

Grundsätzlich kann die autonome Neuropathie alle Organsysteme betreffen. Klinisch bedeutsam sind die gestörte Hypoglykämiewahrnehmung, das Fehlen von Schmerzen bei myokardialer Ischämie (stummer Myokardinfarkt), die Ruhetachykardie und orthostatische Hypotonie sowie die gestörte Magenentleerung mit entsprechend schwieriger glykämischer Kontrolle [7].

Für Betroffene besonders belastend sind urologische Manifestationen der autonomen Polyneuropathie wie die Zystopathie und die erektile Dysfunktion [7]. Die diabetische Zystopathie mit einer Störung der Blasenentleerung kann Anlass für wiederholte Infekte sein, die aufgrund der Sensibilitätsstörungen kaum oder nicht wahrgenommen werden.

Die nachfolgende Tabelle gibt eine Übersicht über verschiedene Organmanifestationen und klinische Bildern der autonomen diabetischen Neuropathie (Tab. 3; [7]):

**Tab. 3:** Klische Bilder der autonomen diabetischen Neuropathie

Kardiovaskuläres System	Ruhetachykardie, reduzierte Herzfrequenzvariabilität, Belastungsintoleranz, perioperative Instabilität, QT-Verlängerung, orthostatische Hypotonie, verminderte bzw. fehlende Wahrnehmung von Myokardischämien
Gastrointestinaltrakt	Dysphagie, gastroösophageale Refluxkrankheit, diabetische Gastropathie (dyspeptische Symptome, postprandiale Hypoglykämie), diabetische Cholezystopathie, diabetische Diarrhö, Hypomotilität von Dünn- und/oder Dickdarm mit Obstipation, chronische intestinale Pseudoobstruktion, anorektale Dysfunktion (meist Stuhlinkontinenz)
Urogenitaltrakt	Diabetische Zystopathie (Harnblasenentleerungsstörung), männliche Sexualstörungen (z. B. erektile Dysfunktion, retrograde Ejakulation), Sexualstörungen der Frau
Neuroendokrines System	Hypoglykämie-assoziierte autonome Dysfunktion (Reduktion bzw. Fehlen der hormonellen Gegenregulation, verminderte Katecholaminsekretion im Stehen und unter körperlicher Belastung, Störung der Hypoglykämiewahrnehmung)
Störungen der Sudomotorik	Dyshidrose, Anhidrose („trockene Füße“), gustatorisches Schwitzen
Vasomotorenstörung	Überwärmte Haut, neuropathisches Ödem, orthostatische Hypotonie
Trophik	Neuropathisches Ulkus, Neuroosteoarthropathie (DNOAP bzw. Charcot-Arthropathie)
Respiratorisches System	Zentrale Fehlregulation der Atmung mit herabgesetztem Atemantrieb gegenüber Hyperkapnie bzw. Hypoxämie, Schlafapnoe, Atemstillstand
Pupillomotorik	Pupillenreflexstörungen, verminderte Dunkeladaption

## Therapie der diabetischen Polyneuropathie (PNP)

- Kausale Therapie: Basis jeder weiteren Behandlung durch optimierte glykämische Kontrolle und Lebensstilintervention.
- Multimodale Schmerztherapie: Kombination von medikamentösen und nichtmedikamentösen Ansätzen.

Die Praxisempfehlungen 2022 der Deutschen Diabetes Gesellschaft empfehlen, erst mit einer medikamentösen Therapie zu beginnen, wenn sich Menschen mit einer diabetisch distalen sensomotorischen Polyneuropathie (DSPN) in ihrem täglichen Leben beeinträchtigt fühlen [8]. Eine medikamentöse Therapie sollte durch nichtmedikamentöse Maßnahmen unterstützt werden.

Die *kausale Therapie* bildet die Grundlage jeder Behandlung [3]. Bereits durch Normoglykämie und angepasste Lebensstilinterventionen können eine Verbesserung der Symptomatik und eine Progressionsvermeidung bei DSPN und kardiovaskulär autonome Neuropathie (CAN) erreicht werden [9, 10]. Der DCCT-Trial zeigte bei Diabetes mellitus Typ 1 eine 60 %ige Reduktion von DSPN und eine 30 %ige Risikoreduktion von CAN durch optimierte glykämische Kontrolle (HbA_1c_ 7,4 % vs. 9,1 %) [2, 11] Der EDIC-Trial bestätigte diese Vorteile auch 13 bis 14 Jahre später, unabhängig von der glykämischen Kontrolle [11, 12]. Bei Diabetes mellitus Typ 2 sind die Ergebnisse weniger eindeutig [13, 14]. Studien wie UKPDS und ACCORD liefern Hinweise auf eine Risikoreduktion für PNP durch intensivierte glykämische Kontrolle, jedoch oft knapp nicht signifikant [12–15]. Gründe hierfür könnten in unterschiedlichen Therapieregimen und womöglich auch in unterschiedlichen Subgruppen des Diabetes mellitus Typ 2 zu finden sein. Potenziell neuroprotektive Substanzen sind SGLT2-Inhibitoren und GLP-1-Rezeptor-Agonisten [16, 17].

Es besteht jedoch Konsens, dass bei allen Diabetestypen Risikofaktoren für die Neuropathie, wie z. B. Rauchen, übermäßiger Alkoholkonsum und assoziierte Begleiterkrankungen (Nephropathie, Retinopathie, KHK, pAVK, Hypertonie, viszerale Adipositas, Dyslipidämie), erfasst und therapiert werden müssen [3].

### Allgemeine Empfehlungen zur Therapie der schmerzhaften PNP

Grundsätzlich stehen systemisch wirksame und topische Therapeutika zur Verfügung, wobei auch eine gleichzeitige Anwendung beider Formen möglich ist [18]. Bei der Therapie der PNP muss dem Behandler bewusst sein, dass eine Schmerzfreiheit oft nicht erreicht werden kann. Dies soll auch dem Patienten so kommuniziert werden, um keine falschen Hoffnungen zu wecken. Bei allen medikamentösen Optionen spricht ein Teil der Menschen nur unzureichend auf die Therapie an oder leidet an nichttolerierbaren Nebenwirkungen [19]. Vor Therapiebeginn sollte zur Verbesserung der Adhärenz über potenzielle Nebenwirkungen aufgeklärt werden. Des Weiteren sollte darüber informiert werden, dass die Wirkung erst nach Auftitration und Erreichen einer wirksamen Dosis und mit Verzögerung einsetzt, um das frühzeitige Absetzten von potenziell wirksamen Präparaten zu vermeiden. Die Wirksamkeit sollte unter ausreichender Dosierung erst nach 2 bis 4 Wochen beurteilt werden. Es kann sinnvoll und effektiver sein, mehrere Medikamente zu kombinieren, da dadurch synergistisch schmerzhemmende Effekte auftreten können und die Einzeldosen niedriger bleiben. Geduld und eine gute Kommunikation zwischen Behandelnden und Behandelten sind wichtig, um die individuell am besten geeignete Therapie zu finden. In die Diagnose und insbesondere auch die Wahl der Therapie sollten Menschen mit Diabetes partizipativ miteinbezogen werden [20]. Eine systemische Pharmakotherapie sollte immer wieder, spätestens alle 3 bis 6 Monate, kritisch reflektiert werden und bei Therapieversagen das Medikament auch wieder abgesetzt werden [18].

Die Wahl des Medikaments richtet sich nach der Wirksamkeit und dem generellen Risikoprofil der Substanzen bei bekannten oder potenziellen Komorbiditäten. Als realistische Therapieziele bei neuropathischen Schmerzen sind in der Regel anzustreben [8, 21, 22]:

- Schmerzreduktion um 30–50 %,
- Verbesserung der Schlafqualität,
- Verbesserung der Lebensqualität,
- Erhaltung der sozialen Aktivität und des sozialen Beziehungsgefüges,
- Erhaltung der Arbeitsfähigkeit, Verbesserung der Funktionalität.

**Abb. 1:**
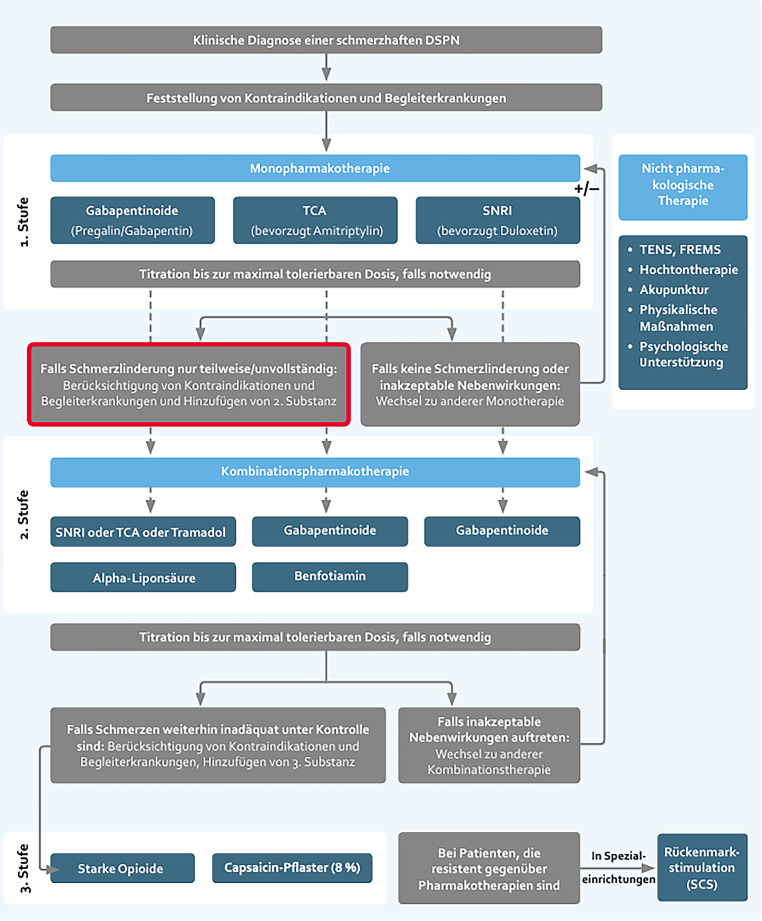
Adaptiert; Konsensusempfehlung: Algorithmus zur Therapieauswahl bei schmerzhafter diabetischer Polyneuropathie in der Praxis [23]

Die *multimodale Schmerztherapie* umfasst neben einer medikamentösen Therapie auch eine topische Therapie, TENS, Psychotherapie, Ergotherapie oder Akupunktur bis hin zu invasiven Verfahren, wie z. B. einer elektrischen Rückenmarkstimulation (SCS) (Abb. 1).

### Medikamentöse Schmerztherapie

(Tab. 4)

**Tab. 4:** Medikamentöse Therapie der schmerzhaften PNP

ARZNEISTOFF	STARTDOSIS	AUFDOSIERUNG ZIELDOSIS (ZD) MAXIMALDOSIS (MTD)	BESONDERHEITEN UND WICHTIGE NEBENWIRKUNGEN
*Medikation 1. WAHL*			
Gabapentin	3 × 100mg bzw. 1 × 300mg (Beginn mit abendlicher Dosis)	Täglich um 300 mg steigern bis 1200 mg/d, dann falls erforderlich wöchentlich um 600 mg steigern ZD 1200–3600 mg/d, 3–4 Dosen MTD 3600 mg/d	Müdigkeit, Schwindel, Gangunsicherheit, periphere Ödeme, kaum Interaktionen, Dosis an Nierenfunktion anpassen, verzögerter Wirkbeginn
Pregabalin	2 × 50–75 mg (Beginn mit abendl. Dosis)	Nach 3–7 Tagen Steigerung um 50–75 mg auf 150 mg/d, dann falls erforderlich wöchentlich um 150 mg steigern ZD 150–600 mg/d, 2 Dosen MTD 600 mg/d	Müdigkeit, Schwindel, Gangunsicherheit, periphere Ödeme, Gewichtszunahme, wirkt anxiolytisch, kaum Interaktionen, Dosis an Nierenfunktion anpassen, verzögerter Wirkbeginn
Amitriptylin	10–25 mg (abends)	10–25 mg alle 7 Tage ZD 25–75 mg/d, bei Depression 75–150 mg/d, Einzeldosis MTD 150 mg/d	Müdigkeit, Schwindel, Sedierung, Sturzgefahr, Miktions- und Akkomodationsstörungen, Hypotonie, Gewichtszunahme, CYP-Interaktionen, langsame Aufdosierung, kardiale NW (EKG Kontrolle)
Duloxetin	30–60 mg (morgens)	30 mg alle 4–7 Tage ZD 60 mg/d morgens MTD 120 mg/d	Übelkeit und Erbrechen, Mundtrockenheit, Blutdruckanstieg, CYP-Interaktionen, Dosisanpassung bei Rauchern, keine Kombi mit Tramadol, Triptanen oder Johanniskraut
*Medikation 2. WAHL*			
Lidocain Pflaster	5 % (700 mg) 1 × tgl. Bis zu 12 h Pause	1–3 Pflaster täglich	Erythem und Unverträglichkeitsreaktionen am Applikationsort, kaum systemische NW
Capsaicin Pflaster	8 % (179 mg) 1 × 30 min; 90 Tage Pause	1–4 Pflaster pro Anwendung alle 3 Monate oder später	Erythem, Rötung, Brennschmerz und Unverträglichkeitsreaktionen am Applikationsort, temporäre Schmerzzunahme ggf. Blutdruckanstieg, keine systemischen Nebenwirkungen oder Medikamentenwechselwirkungen
Tramadol retard	2–3 × 50–100 mg	50–100 mg alle 3–4 Tage ZD 100–200 mg/d, 2–3 Dosen MTD 600 mg/d	Übelkeit, Hypotonie, Dosisreduktion bei eingeschränkter Nierenfunktion, keine Kombi mit serotonergen Substanzen oder Duloxetin
Tapentadol retard	2 × 50 mg	100 mg alle 3–4 Tage ZD 100–200 mg/d, 2–3 Dosen MTD 500 mg/d	Übliche Opioid-NW bei geringerer Obstipation und Absetzproblematik
*Medikation 3. WAHL*			
Carbamazepin	100–400 mg (abends)	200 mg alle 3–7 Tage ZD 600 mg/d, 2 Dosen MTD 1200 mg/d	Kognitive Beeinträchtigung, Blutbildveränderungen, Leberschäden, Hyponatriämie, Hautausschlag, Medikamenteninteraktionen wegen Enzyminduktion, langsame Aufdosierung nötig
Oxycodon retard	2–3 × 5–10 mg	Individuell MTD Tumorpat. 400 mg/d	Übliche Opioid-NW, Dosisreduktion bei Leber- und Niereninsuffizienz
Buprenorphin TTS	5–20 µg/h	Individuell	Übliche Opioid-NW, keine Dosisreduktion bei eingeschränkter Nierenfunktion
Botolinumtoxin	–	–	–

Topische Therapie: Lokale Anwendungen von Capsaicin-Cremes oder hoch dosierten Capsaicin-Pflastern können zur Schmerzlinderung beitragen [19, 24]. Die topische Therapie kann die orale Therapie gut unterstützen.

Alpha-Liponsäure und Benfotiamin können als pathogenetisch orientierte Therapie ergänzend oder bei nicht schmerzhafter Dysästhesie verordnet werden. In mehreren kleinen Studien und Metaanalysen konnte ein positiver Effekt mit den genannten Substanzen bei DSPN gezeigt werden [3, 21, 25–32, 37]. Allerdings ist zu sagen, dass die Evidenzlage nicht optimal und die Therapie kontrovers diskutiert wird. Es wäre sehr wünschenswert, wenn neue Studien mit mehr Teilnehmern und einem längeren Beobachtungszeitraum durchgeführt werden würden, um die positive Wirkung bestätigen zu können [32]. Zu den offenen Fragen gehört, ob diese Therapie präventiv wirkt und wie lange sie erfolgen sollte [3]. In einer Metaanalyse wurden erniedrigte systemische Spiegel von Thiamin (Vitamin B_1_) bei Menschen mit Diabetes nachgewiesen, sodass davon ausgegangen werden darf, dass bei Menschen mit Typ-1- als auch bei Menschen mit Typ-2-Diabetes ein erhöhter Thiaminbedarf besteht [33, 34].

Physiotherapie und Rehabilitation können dazu beitragen, Bewegungseinschränkungen und Schmerzen zu reduzieren [35].

Transkutane elektrische Nervenstimulation (TENS): TENS-Geräte verwenden elektrische Impulse, um die Schmerzsignale zu stören und die Schmerzen zu lindern. Die Evidenz ist jedoch niedrig [36].

Psychotherapie: Bei neuropathischen Schmerzen können eine psychologische Unterstützung und Therapie hilfreich sein, um den Umgang mit Schmerzen und die Bewältigung von Stress zu verbessern [19].

### Therapiealternativen bei refraktärem Schmerz

Bei Nichtansprechen der oben genannten Therapien stehen noch Baclofen, intravenöses Lidocain oder eine Rückenmarkstimulation zur Verfügung. Insbesondere der Letzteren sollte ein interdisziplinäres Assessment vorgeschaltet sein, um den biopsychosozialen Ursachen von Schmerzen gerecht zu werden.

## Diabetisches Fußsyndrom

Das diabetische Fußsyndrom gehört zu den schwerwiegendsten Folgeerkrankungen des Diabetes mellitus. Definiert ist das diabetische Fußsyndrom als Ulzeration, Infektion oder Gewebedestruktion an der unteren Extremität bei Menschen mit Diabetes mellitus, verursacht durch eine Neuropathie und/oder peripher arterielle Verschlusskrankheit. 19–34 % aller Menschen mit Diabetes entwickeln im Laufe der Erkrankung ein Ulkus [38]. Es ist mit 40–60 % die Hauptursache für nichttraumatische Amputationen der unteren Extremität [39].

## Pathophysiologie

Die Pathogenese des diabetischen Fußsyndroms ist multifaktoriell. 90 % aller Menschen mit Diabetes mellitus und diabetischem Fußsyndrom haben eine sensomotorische Neuropathie. Eine peripher arterielle Verschlusskrankheit liegt bei mindestens 50 % der Fälle vor [40]. Der Verlust der Warn‑/Schutzfunktion durch die eingeschränkte oder fehlende Sensibilität („loss of protective sensation“ [LOPS]), Fußdeformitäten und eine eingeschränkte Mobilität der Gelenke führt zu einer abnormalen biomechanischen Belastung des Fußes. Dieser erhöhte Druck führt zur Bildung von Hyperkeratosen (Kallus) mit möglicher Blasenbildung oder Einblutung und daraus resultierenden Ulzerationen. Neben der abnormen biomechanischen Belastung mit konsekutiven Ulzerationen sind Mikrotraumata eine weitere häufige Ursache von Ulzerationen. Mikrotraumata entstehen meist durch nicht passendes Schuhwerk oder eine falsche Fußpflege (z. B. Verletzung beim Kürzen der Nägel). Auch thermische Verletzungen sind eine mögliche Ursache von Ulzerationen, z. B. Verbrennungen durch heiße Oberflächen/Wasser oder Erfrierungen.

Die Neuropathie selbst führt zu einer Körperwahrnehmungsstörung und einem Neglect. Diese bestehende psychologische Komponente begünstigt die Entstehung des diabetischen Fußsyndroms und erschwert besonders die Behandlung, da Patient:innen ihr Problem nicht wahrnehmen können.

Die periphere arterielle Verschlusskrankheit (PAVK) ist ein wichtiger Risikofaktor für eine schlechte Wundheilung und ein hohes Amputationsrisiko. Bei Menschen mit neuroischämischen Ulzerationen ist zu beachten, dass meist die klassische Symptomatik der Claudicatio intermittens aufgrund der bestehenden Neuropathie fehlt. Daher muss jede Ulzeration beim Menschen mit Diabetes weiterführend angiologisch abgeklärt werden. Das für den individuellen Menschen optimale bildgebende Verfahren wird in Abhängigkeit von Komorbiditäten wie einer Niereninsuffizienz und lokaler Expertise gewählt [41].

## Prävention

Primäres Ziel bei der Behandlung von Menschen mit Diabetes ist die Prävention von Spätkomplikationen durch eine optimale Stoffwechselkontrolle und Optimierung der kardiovaskulären Risikofaktoren.

Zur Prävention des diabetischen Fußsyndroms gibt es 5 Schlüsselelemente:

1. Identifikation eines Risikofußes,
2. regelmäßige Inspektion und Untersuchung des Risikofußes,
3. Schulung des Menschen mit Neuropathie, dessen Angehörige und von im Gesundheitsbereich arbeitenden Personen,
4. Tragen von geeignetem Schuhwerk,
5. Behandlung von präulzerösen Läsionen wie Hornhautschwielen.

Die Identifikation eines Risikofußes erfolgt nach Tab. 5. Eine fehlende protektive Wahrnehmung („loss of protective sensation“ [LOPS]) wird im Regelfall durch die Anamnese, klinische Untersuchung und durch ein Neuropathie-Screening diagnostiziert. Bei fehlenden Ressourcen kann das Neuropathie-Screening auf das Druck- bzw. Berührungsempfindung mit dem 10 g-Monofilament reduziert werden. Neurophysiologische Untersuchungen sind nur in Ausnahmefällen notwendig. Liegt eine bekannte Neuropathie, PAVK, Fußdeformität oder ein stattgehabtes Ulkus/eine Amputation vor, besteht immer ein erhöhtes Ulkusrisiko. Ein Neuropathie-Screening muss dann nicht mehr durchgeführt werden. Die Füße müssen jedoch regelmäßig von medizinischem Fachpersonal inspiziert werden. Hinsichtlich einer möglichen PAVK sollten bei vermeintlich gesundem Fuß zumindest einmal jährlich eine Überprüfung der Fußpulse stattfinden.

**Tab. 5:** Risikostratifizierung für ein Ulkus am Fuß bei Menschen mit Diabetes. (Adaptiert nach www.Iwgdf.org; Mit freundlicher Genehmigung von Universimed)

Kategorie	Ulkusrisiko	Charakteristik	Fußkontrolle^a^
0	Sehr niedrig	Keine fehlende protektive Wahrnehmung	Einmal jährlich
1	Niedrig	Fehlende protektive Wahrnehmung *oder* Periphere Durchblutungsstörung	Alle 6–12 Monate
2	Moderat	Fehlende protektive Wahrnehmung und periphere Durchblutungsstörung *oder* Fehlende protektive Wahrnehmung und Fußdeformation *oder* Periphere Durchblutungsstörung und Fußdeformation	Alle 3–6 Monate
3	Hoch	Fehlende protektive Wahrnehmung oder periphere Durchblutungsstörung und ein oder mehrere folgende Faktoren:	Alle 1–3 Monate
		Früherer Fußulkus	
		Amputation unterer Extremität (klein oder groß)	
		Terminale Niereninsuffizienz	

Die Kontroll-Intervalle nach Tab. 5 basieren auf Expertenmeinung. Aufgrund des Neglects für ihre Erkrankung ist es von Vorteil, wenn bei der Schulung bzw. Aufklärung über das diabetische Fußsyndrom Angehörige einbezogen werden. Präventiv sollten die Füße einmal täglich inklusive Fußsohle und Zehenzwischenräume kontrolliert werden. Die Hautpflege erfolgt mit ureahaltigen Pflegecremes. Damit kann die Widerstandsfähigkeit der Haut gegenüber Traumen erhöht werden. Geeignetes Schuhwerk ist zur Prävention essenziell. Prinzipiell neigen Menschen mit Neuropathie dazu, zu enges Schuhwerk zu bevorzugen, da sie das Gefühl haben, damit besser gehen zu können. Ist die Fußdeformität nicht zu ausgeprägt, sind Konfektionsschuhe mit Weichbettungseinlagen zur Primärprävention geeignet. Bei einer ausgeprägten Fußdeformität müssen orthopädische Maßschuhe angepasst werden. Vor dem Anziehen der Schuhe ist der Schuh auf Fremdkörper zu überprüfen. Zu vermeiden ist barfuß oder mit Socken zu gehen, da Fremdkörper zu Verletzungen führen können. Ein zu heißes Fußbad ist neben heißen Oberflächen wie Sand eine häufige Ursache von Verbrennungen. Auf eine korrekte und verletzungsfreie Nagelpflege ist zu achten [40]. Als präulzeröse Läsionen bezeichnet man Hyperkeratosen oder Nagelfehlstellungen. Insbesondere Hyperkeratosen müssen regelmäßig entfernt werden. Dies sollte durch eine professionelle Fußpflege erfolgen. Aufgrund des häufig vorhandenen sozioökonomischen Nachteils von Menschen mit Diabetes ist dies nicht immer möglich.

Bei der klinischen Untersuchung von Füßen in der Risikokategorie 1–3 sollte auf Folgendes geachtet werden:

1. Hautfarbe, Temperatur, Hornhautschwielen, Ödeme, Muskelatrophien
2. Fußdeformitäten wie Krallenzehen, verändertes Längs- und Quergewölbe, prominenter Mittelfußbereich, abnorme Knochenvorsprünge
3. Ist korrektes Schuhwerk vorhanden?
4. Adäquate Fußhygiene und Fußpflege
5. Wissen zum diabetischen Fußsyndrom

## Evaluierung von Fußulzerationen

Das diabetische Fußsyndrom sollte standardisiert evaluiert werden, um eine optimale Therapie zu ermöglichen.

### Klassifikation von Ulzerationen

Es gibt verschieden Scores, um Ulzerationen bei Menschen mit Diabetes zu klassifizieren. Von der IWGDF (International Working Group on the Diabetic Foot) [42] wird der SINBAD-Score empfohlen, da er einfach anzuwenden ist (Tab. 6). In einer multizentrischen Studie zeigte sich eine deutliche Verlängerung in der Zeit bis zur Abheilung bei einem SINBAD-Score von 2–3. Bei einem Score von 3 und mehr ist eine Abheilung oft unwahrscheinlich [42].

**Tab. 6:** SINBAD Klassifikation

Kategorie	Definition	Punkte
Ulkuslokalisation	Vorfuß	0
	Mittelfuß oder Rückfuß	1
Ischämie	Zumindest ein Puls tastbar	0
	Klinischer Hinweis auf Durchblutungsstörung	1
Neuropathie	Keine Neuropathie	0
	Neuropathie	1
Infektion	Keine Infektion	0
	Infektion	1
Ulklusgröße	Ulkus keiner 1 cm^2^	0
	Ulkus größer 1 cm^2^	1
Ulkustiefe	Oberflächliches Ulkus	0
	Ulkus mit Sehen oder Muskelbeteiligung oder tiefer	1
Maximale Punktezahl		6

Durch die klinische Untersuchung kann zumindest teilweise ein Ulkus einer im Vordergrund stehenden Pathophysiologie zugeordnet werden. Ein neuropathisches Ulkus präsentiert sich an bekannten Prädilektionsstellen vor allem im Bereich der Köpfe der Os metatarsalia oder an den Zehenspitzen bzw. dorsal an den Interphalangealgelenken. Die Ulzerationen sind meist rund, wie ausgestanzt mit einer umgebenden Hyperkeratose. Die Hyperkeratose ist Ausdruck einer Druckbelastung. Das ischämische Ulkus ist meist akral lokalisiert im Sinne einer Nekrose. Da eine ischämische Komponente häufig ist, muss auch bei neuropathisch imponierenden Wunden immer eine angiologische Abklärung erfolgen.

### Infektion

**Abb. 2:**
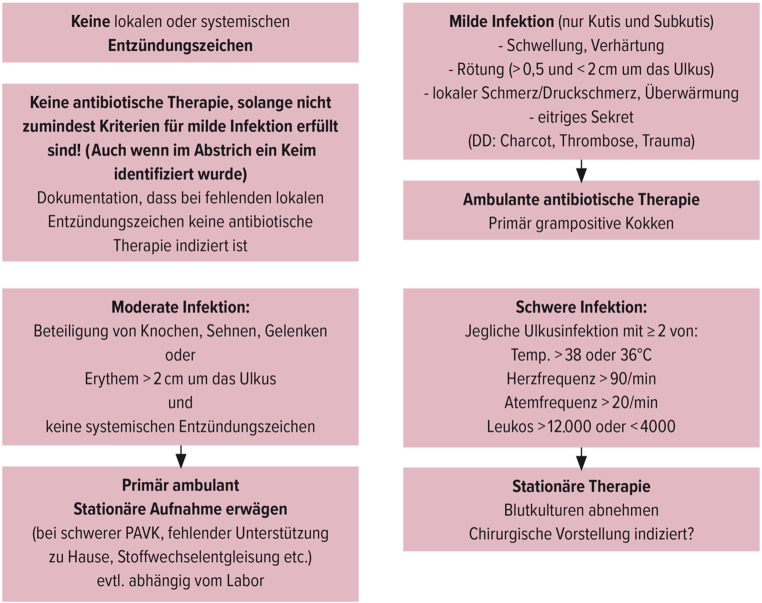
Einteilung von Infektionen beim diabetischen Fußsyndrom. (Mit freundl. Genehmigung von Universimed, adaptiert nach https://iwgdfguidelines.org; Körpertemperatur > 38 oder < 36 = Körpertemperatur)

Jede Infektion kann den Fuß des Menschen mit Diabetes gefährden. Daher müssen Ulzerationen regelmäßig klinisch auf das Vorhandensein einer Infektion evaluiert werden. Da alle Ulzerationen mit Bakterien kolonisiert sind, basiert die Diagnose einer Infektion auf dem Vorhandensein von mindestens 2 entsprechenden klinischen Zeichen (Rötung, Schwellung, Überwärmung, Schmerz). Sind Infektionszeichen vorhanden erfolgt die Einteilung der Infektion nach Abb. 2. Infektionen müssen prompt therapiert werden.

### Osteomyelitis (OM)

Infektionen des Knochens per continuitatem am Fuß treten häufig auf. Die knöchernen Strukturen liegen nahe an der Oberfläche. Die Diagnose gestaltet sich immer wieder schwierig, da eindeutige Standards fehlen. Bei Ulzerationen ist eine „probe-to-bone“ obligatorisch. Mit einer Sonde oder ähnlichem Instrument wird die Wunde sondiert und geprüft, ob der Knochen tastbar ist. Ist kein Knochen tastbar, ist eine Osteomyelitis eher unwahrscheinlich. Die „probe-to-bone“ ist von der Erfahrung des Untersuchers abhängig und daher schwer reproduzierbar.

Besteht der Verdacht auf eine OM ist der nächste diagnostische Schritt ein konventionelles Röntgen, da kostengünstig und überall verfügbar. Kann im Röntgen die Diagnose nicht bestätigt werden, aber der Verdacht auf eine Knochenbeteiligung besteht weiterhin, ist die Magnetresonanzuntersuchung das Mittel der Wahl mit der höchsten Spezifität und Sensitivität [42].

## Therapie

Aufgrund seiner Komplexität benötigt das diabetische Fußsyndrom immer einen multiprofessionellen Behandlungsansatz.

### Therapieziel

Die Definition eines auf das Individuum zugeschnittenen Therapieziels abhängig von verschiedenen Faktoren wie Begleiterkrankungen und der Lebenserwartung ist essenziell. Neben der Abheilung einer Ulzeration kann auch die Erhaltung der Mobilität bzw. eine Amputations- und Infektionsvermeidung im Vordergrund stehen.

### Druckentlastung

Der Grundpfeiler in der Behandlung von Ulzerationen, welche durch vermehrten biomechanischen Stress verursacht wurden, ist die Druckentlastung der Läsion. Ist das Therapieziel die Abheilung der Läsion, ist dies die wichtigste und zugleich im täglichen Leben am schwersten umsetzbare therapeutische Maßnahme.

**Abb. 3:**
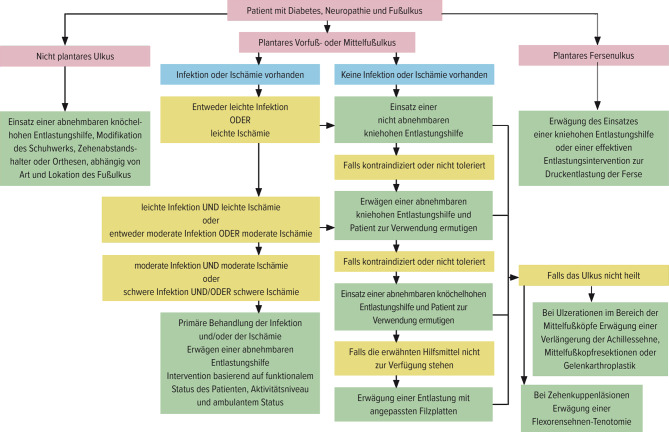
Druckentlastung beim diabetischen Fußsyndrom. (Adaptiert nach https://iwgdfguidelines.org; mit freundl. Genehmigung durch Universimed)

Es gilt der Leitsatz: „Hit hard and early“. Der Goldstandard bei der Entlastung ist ein Vollkontaktgips. Seine Überlegenheit wurde in mehreren randomisiert kontrollierten Studien nachgewiesen. Dieser garantiert eine Druckentlastung 24 h am Tag. Wird dieser vom Menschen mit einem DFS nicht akzeptiert oder toleriert sollte eine kniehohe abnehmbare Vakuumschiene (z. B. VACO®CastOped AG, Steinhausen, Schweiz) in Betracht gezogen werden. In der Step-down-Therapie folgen als Empfehlung anpassbare Verbandsschuhe (z. B. WCS® Verbandschuh, Darco Europe, Raisting, Deutschland). Eine individualisierte Druckentlastung kann nach Abb. 3 erfolgen. Jegliche Form von Vorfußentlastungsschuhen ist aufgrund der Sturzgefahr obsolet.

### Periphere arterielle Verschlusskrankheit

Nach weiterführender stadiengerechter Diagnostik und konklusiver Darstellung der arteriellen Strombahn (Duplexsonographie, MRA, CTA) gilt die Revaskularisation als zentrales Ziel bei vorhandenen Stenosen. Der optimale Behandlungsmodus (endovaskulär vs. operativ vs. Hybridverfahren) sollte in einem interdisziplinären Gefäßboard festgelegt werden.

### Infektion

Milde und moderate Infektionen werden im Regelfall 2 Wochen und eine schwere Infektion 3 Wochen antibiotisch je nach vorhandener Resistenzlage therapiert. Wenn möglich, sollte immer ein Keimnachweis angestrebt werden. Eine Gewebeprobe ist einem tiefen Wundabstrich vorzuziehen. Bei einer milden Infektion sollte eine empirische Antibiose mit Substanzen gegen *Staphylokokkus aureus* und Streptokokken begonnen werden. Bei moderaten bis schweren Infektionen muss sowohl das grampositive als auch das gramnegative Spektrum an möglichen Keimen abgedeckt werden. Gegebenenfalls muss auch eine chirurgische Nekrektomie evaluiert werden [43].

Die Therapiedauer einer Knocheninfektion liegt in der Regel bei 6 Wochen. Hier stellt aus infektiologischer Sicht eine Knochenbiopsie den diagnostischen Goldstandard dar. Da diese jedoch oft mangels Know-how und Ressourcen nicht umsetzbar ist, sollte zumindest eine tiefe Gewebebiopsie erfolgen. Bei einem *S. aureus* in der tiefen Gewebebiopsie kann wahrscheinlich auf eine Knochengewinnung verzichtet werden [44]. Bei komplexen Fragestellungen sollte mit Infektiolog:innen Kontakt aufgenommen werden.

Führt eine antibiotische Therapie nicht zum Erfolg, muss gegebenenfalls eine chirurgische Sanierung in Betracht gezogen werden.

### Amputationen

Amputationen sollten immer vermieden werden, und es ist zu überlegen, ob vor Amputationen nicht das Therapieziel angepasst werden soll. Es muss bedacht werden, dass selbst Minor-Amputationen durch eine veränderte Biomechanik am Fuß wieder zu neuen Ulzerationen (Transferulkus) führen können.

### Lokaltherapie

Die feuchte Wundbehandlung gehört zu den Standards bei der Behandlung von Ulzerationen. Die Evidenz von Vorteilen einzelner Verbandsstoffe ist eingeschränkt.

Primär gilt, dass die Verbandsauswahl je nach Wundstadium und Exsudation erfolgt. Trockene Nekrosen sollten trocken gehalten werden. Vorhandene Hyperkeratosen, Beläge und nekrotisches Gewebe sollten mechanisch entfernt werden. Beim scharfen Débridement zeigte eine Studie einen Vorteil gegenüber einer Standardtherapie. Ein enzymatisches Débridement brachte im Vergleich zu Hydrogel keinen Vorteil. Studien zur Madentherapie sind mit einem hohen Bias verbunden, und somit ist keine Beurteilung hinsichtlich eines Zusatznutzens möglich [43]. Auch Wundauflagen mit Wirkstoffen inklusive Silber zeigten keinen eindeutigen vorteilhaften Nutzen. Ein Cochrane Review aus dem Jahr 2017 bestätigt lediglich, dass antimikrobielle Wundauflagen die Heilungsrate verbessern könnten. Der Evidenzgrad ist jedoch niedrig [45].

Eine Wundauflage mit einer Sucrose-Octasulfat-Imprägnierung zeigte bei nicht infizierten neuroischämischen Fußulzerationen einen signifikanten Vorteil [46]. Biotechnologische Ansätze wie Hautersatz oder allogenes plättchenreiches Plasma zeigten Vorteile bei geringer Qualität der Evidenz [47]. Die Stoßwellentherapie zeigte ebenfalls in einer Metaanalyse einen leichten Vorteil gegenüber der Standardtherapie [48]. Die hyperbare Sauerstoff(HBO)-Therapie wird ebenfalls kontroversiell diskutiert und kann daher in Spezialfällen zur Anwendung kommen. Eine VAC-Therapie ist bei postoperativen Wunden ebenfalls erwägbar. Aufgrund der geänderten Datenlage zur Anwendung von topischem Sauerstoff kann diese als adjuvante Therapie bei fehlender Wundheilung unter Standardtherapie empfohlen werden. Jedoch ist die Verfügbarkeit dieser Systeme begrenzt.

## Diabetische Neuroosteoarthropathie – Charcot-Fuß

Die diabetische Charcot-Osteoarthropathie (diabetische Neuroosteoarthropathie [DNOAP]) ist die komplexeste und schwerwiegendste Fußkomplikation. Es handelt sich um eine destruierende Erkrankung des Fußskeletts infolge einer sensorischen und autonomen Neuropathie, die zu Knochen- und einer Gelenkzerstörung führt. Die Ätiopathogenese ist nicht im Detail geklärt. Die Inzidenz liegt bei 0,3 % pro Jahr. Ein Charcot-Fuß präsentiert sich in der akuten Phase als rot, geschwollen und überwärmt. Es besteht eine mehr oder weniger ausgeprägte Fußdeformität durch das Auftreten von Spontanfrakturen [49]. Zehn bis fünfzehn Prozent der Betroffenen klagen über Schmerzen. In der akuten Phase der Erkrankung besteht immer die Gefahr, dass die Fußdeformität voranschreitet oder es durch das Abkippen von Knochenfragmenten zu chronischen Ulzerationen kommt. Dadurch ist die Amputationsgefahr hoch. Die diabetische Neuroosteoarthropathie tritt meist im Mittelfußbereich auf, kann aber auch an anderen Lokalisationen wie z. B. im Sprunggelenk manifestieren.

Die Diagnose erfolgt primär klinisch (Wärmeunterschied > 2 °C im Seitenvergleich; Schwellung, Rötung, Fußdeformität).

Die Bildgebung wie Röntgen oder MR kann bei der Diagnose zu Problemen führen. Ziel einer Behandlung ist es, den akuten Charcot-Fuß in eine chronisch inaktive Form zu überführen. Beim chronisch inaktiven Charcot-Fuß kann davon ausgegangen werden, dass der Fuß wieder belastbar ist, ohne dass es zu weiteren Fußdeformitäten kommt.

Die einzige derzeit anerkannte Behandlungsoption ist eine Druckentlastung für 6 bis 12 Monate, welche im Regelfall mit einem Vollkontaktgips durchgeführt wird. Eine medikamentöse Therapie mit Bisphosphonaten, Calcitonin, PTH, Kortikosteroiden oder Denosumab wird nicht empfohlen. Eine Vitamin-D- und Kalzium-Supplementierung kann jedoch bei entsprechendem Mangel erwogen werden, insbesondere in der Phase der Frakturheilung.

Das Vorliegen eines chronisch inaktiven Charcot-Fußes kann angenommen werden, wenn die Rötung und das Ödem sich zurückgebildet haben und der Temperaturunterschied zum nicht betroffenen Fuß unter 2 °C liegt. Der Fuß kann dann mit einem orthopädischen Maßschuh zur Verhinderung von Ulzerationen versorgt werden. Rezidive oder das Auftreten an der kontralateralen Extremität sind keine Seltenheit.

Merksatz: Jeder heiße, rote, geschwollene Fuß bei Patient:innen mit Neuropathie ist ein Charcot-Fuß – bis das Gegenteil bewiesen ist.

Ein Charcot-Fuß sollte immer in einem Zentrum mit entsprechender Expertise versorgt werden.

## References

[1] -. Nationale Versorgungsleitlinie: Neuropathie bei Diabetes im Erwachsenenalter, Langversion. 1. Aufl. 2011. 10.1007/BF03369208 27371070

[2] Ziegler D, Papanas N, Vinik AI, Shaw JE. Epidemiology of polyneuropathy in diabetes and prediabetes. Handb Clin Neurol. 2014;126:3–22. 10.1016/b978-0-444-53480-4.00001-1. 25410210 10.1016/B978-0-444-53480-4.00001-1

[3] Ziegler D. Update: Diabetische Neuropathie erfolgreich diagnostizieren und therapieren. Rheinland-Pfalz: Akademie für Ärztliche Fortbildung; 2024.

[4] Thomas PK, Tomlinson DR. Diabetic and Hypoglycemic Neuropathy. In: Dyck PJ, Thomas PK, Hrsg. Peripheral Neuropathy. 3. Aufl. Philadelphia: Saunders; 1993. S. 1219–50.

[5] Callaghan BC, Cheng HT, Stables CL, Smith AL, Feldman EL. Diabetic neuropathy: clinical manifestations and current treatments. LancetNeurol. 2012;11(6):521–34. 10.1016/S1474-4422(12)70065-0 PMC4254767 22608666

[6] Feldman EL, Callaghan BC, Pop-Busui R, Zochodne DW, Wright DE, Bennett DL, et al. Diabetic neuropathy. Nat Rev Disprimers. 2019;5(1):41. 10.1038/s41572-019-0092-1 31197153

[7] Ziegler D. Diagnosis and treatment of diabetic autonomic neuropathy. CurrDiabRep. 2001;1(3):216–27. 10.1007/s11892-001-0037-3 12643202

[8] Kellerer M, et al. Praxisempfehlungen der Deutschen Diabetes Gesellschaft. Diabetologie. 2022;17(2):S339–S53. 10.1055/a-1916-2156.

[9] Dyck PJ, Karnes JL, O’Brien PC, Litchy WJ, Low PA, Melton LJ. The Rochester Diabetic Neuropathy Study: Reassessment of tests and criteria for diagnosis and staged severity. Neurology. 1992;42(6):1164–70. 1603343 10.1212/wnl.42.6.1164

[10] Dyck PJ, Kratz KM, Karnes JL, Litchy WJ, Klein R, Pach JM, et al. The prevalence by staged severity of various types of diabeticneuropathy, retinopathy, and nephropathy in a population-based cohort: the Rochester Diabetic Neuropathy Study. Neurology. 1993;43(4):817–24. 8469345 10.1212/wnl.43.4.817

[11] Van de Van de Poll-Franse LV, Van de van Valk GD, Renders CM, Heine RJ, van van Eijk JTM. Longitudinal assessment of the development of diabeticpolyneuropathyandassociatedrisk factors. Diabet Med. 2002;19(9):771–6. 12207815 10.1046/j.1464-5491.2002.00778.x

[12] Young MJ, Boulton AJ, MacLeod AF, Williams DR, Sonksen PH. A multicentre study of the prevalence of diabetic peripheral neuropathy in the United Kingdom hospital clinicpopulation. Diabetologia. 1993;36(2):150–4. 8458529 10.1007/BF00400697

[13] Maser RE, Steenkiste AR, Dorman JS, Nielsen VK, Bass EB, Manjoo Q, et al. Epidemiological correlates of diabetic neuropathy. Rep From Pittsburgh Epidemiol Diabetes Complicat Studydiabetes. 1989;38(11):1456–61. 10.2337/diab.38.11.1456 2620781

[14] Tesfaye S, Selvarajah D. Advances in the epidemiology, pathogenesis and management of diabetic peripheral neuropathy. DiabetesMetabResRev. 2012;28(1):8–14. 10.1002/dmrr.2239 22271716

[15] The Look AHEAD Research Group. Effects of a long-term lifestyle modification programme on peripheral neuropathy in overweight or obese adults with type 2 diabetes: the Look AHEAD study. Diabetologia. 2017;60:980–8. 28349174 10.1007/s00125-017-4253-z PMC5423967

[16] Kandeel M, et al. The outcomes of Sodium-Glucose Co-transporter 2 inhibitors (SGLT2I) on Diabetes-Associated Neuropathy: A Systematic Review an meta-Analysis. 13:926717. 10.3389/fphar.2022.926717. 10.3389/fphar.2022.926717 PMC9310020 35899123

[17] Dhanapalaratnam R, et al. Impact of glucagon-like peptide‑1 receptor agonists on axonal function in diabetic peripheral neuropathy. JNP. 2024; 10.1152/jn.00228.2024. 39584713 10.1152/jn.00228.2024

[18] Gillhausen K, et al. Diagnose und Therapie neuropathischer Schmerzen: eine praktische Hilfe für den hausärztlichen Alltag https://docplayer.org/123308213-Diagnose-undtherapie-neuropathischer-schmerzen.html. Zugegriffen: 30. Okt. 2022.

[19] Schlereth T. Diagnose und nicht interventionelle Therapie neuropathischer Schmerzen, S2k-Leitlinie, 2019. Deutsche Gesellschaft für Neurologie https://dgn.org/leitlinien. Zugegriffen: 30. Okt. 2022.

[20] Graggober G, et al. Chronische periphere neuropathische Schmerzen: Diagnose und Therapie in der Praxis. SchmerzNachrichten; 2020.

[21] Ziegler D, et al. Diabetische Neuropathie. Diabetol Stoffwechs. 2024;19:S322–S36. 10.1055/a-2312-0661.

[22] Rittner H. Behandlung von Schmerzen bei peripherer diabetischer Polyneuropathie. Arzeiverordnung in der Praxis/Band 50/Heft 3 https://www.akdae.de/arzneimitteltherapie/arzneiverordnung-in-der-praxis/ausgaben-archiv/ausgaben-ab.

[23] Ziegler D, et al. Screening, diagnosis and management of diabetic sensorimotor polyneuropathy in clinical practice: International expert consensus recommendations. Diabetes Res Clin Pract. 2022;186:109063. 34547367 10.1016/j.diabres.2021.109063

[24] van Nooten F, Treur M, Pantiri K, Stoker M, Charokopou M. Capsaicin 8 % patch versus oral neuropathic pain medications for the treatment of painful diabetic peripheral neuropathy: a systematic literature review and network meta-analysis. ClinTher. 2017;39(4):787–803.e18. 10.1016/j.clinthera.2017.02.010 28365034

[25] Ziegler D, et al. Treatment of symptomatic diabetic polyneuropathy with the antioxidant alpha-lipoic acid: a meta-analysis. Diabet Med. 2004;21(16):114–21. 14984445 10.1111/j.1464-5491.2004.01109.x

[26] Mijnhout GS, et al. Alpha Lipoic Acid for Symptomatic Peripheral Neuropathy in Patients with Diabetes: A Meta-Analysis of Randomized Controlled Trials. Int J Endocrinol. 2012;2012(17):1–8. 10.1155/2012/456279 PMC3272801 22331979

[27] Çakici N, et al. Systematic review of treatments for diabetic peripheral neuropathy. Diabet Med. 2016;33(18):1466–76. 26822889 10.1111/dme.13083

[28] Dy S, et al. Preventing Complications and Treating Symptoms of Diabetic Peripheral Neuropathy. Rockv. 2017;19. 28749633

[29] Cassanego G, et al. Evaluation of the analgesic effect of α‑lipoic acid in treating pain disorders: A systematic review and meta-analysis of randomized controlled trials. Pharmacol Res. 2022;177(106075):20. 10.1016/j.phrs.2022.106075 35026405

[30] Hsieh R‑Y, et al. Effects of Oral Alpha-Lipoic Acid Treatment on Diabetic Polyneuropathy: A Meta-Analysis and Systematic Review. Nutrients. 2023;15. 10.3390/nu15163634 PMC10458197 37630823

[31] Ziegler D, et al. Efficacy and safety of antioxidant treatment with α‑lipoic acid over 4 years in diabetic polyneuropathy: the NATHAN 1 trial. Diabetes Care. 2011;34:2054–60. 21775755 10.2337/dc11-0503 PMC3161301

[32] Stracke H, et al. Exp Clin Endocrinol Diabetes. 2008;116(10):600–5. 10.1055/s-2008-1065351. 18473286 10.1055/s-2008-1065351

[33] Ziegler D, et al. Association between diabetes and thiamine status—A systematic review and meta-analysis. Metabolism. 2023;144:155565. 37094704 10.1016/j.metabol.2023.155565

[34] Thornalley PJ, et al. High prevalence of low plasma thiamine concentration in diabetes linked to a marker of vascular disease. Diabetologia. 2007;50:2164–70. 17676306 10.1007/s00125-007-0771-4 PMC1998885

[35] Baron R, Koppert W, Strumpf M, Willweber-Strumpf A. Interdisziplinäre Diagnostik – multimodaleTherapie. In: Praktische Schmerzmedizin. 4. Aufl. Berlin, Heidelberg: Springer; 2019.

[36] Gibson W, Wand BM, O’Connell NE. Transcutaneous electrical nerve stimulation (TENS) for neuropathic pain in adults. Cochrane Database Syst Rev. 2017;9:CD11976. 10.1002/14651858.CD011976.pub2 PMC6426434 28905362

[37] Gleiter C, et al. Influence of food intake on the bioavailability of thioctic acid entantiomers. Eur J Clin Pharmacol. 1996;50(6):513–4. 10.1007/s00228005015181. 8858282 10.1007/s002280050151

[38] Ziegler D, Gries FA, Spüler M, Lessmann F. The epidemiology of diabetic neuropathy. Diabetic Cardiovascular Autonomic Neuropathy Multicenter Study Group. J Diabetescomplications. 1992;6(1):49–57. 10.1016/1056-8727(92)90049-q 1562759

[39] ADVANCE Collaborative Group, Patel A, MacMahon S, Chalmers J, Neal B, Billot L, et al. Intensive blood glucose control and vascular outcomes in patients with type 2 diabetes. NEngl Jmed. 2008;358(24):2560–72. 10.1056/NEJMoa0802987 18539916

[40] Singh N, Armstrong DG, Lipsky BA. Preventing footulcersin patientswithdiabetes. JAMA. 2005;293(2):217–28. 15644549 10.1001/jama.293.2.217

[41] Aboyans V, Ricco JB, Bartelink MLEL, Björck M, Brodmann M, Cohnert T, et al. 2017 ESC Guidelines on the diagnosis and treatment of peripheral arterial diseases, in collaboration with the European Society for Vascular Surgery (ESVS). Eur Heart J. 2018;39(9):763–816. 29425606 10.1016/j.rec.2017.12.014

[42] Schaper NC, van Netten JJ, Apelqvist J, Bus SA, Fitridge R, Game F, Monteiro-Soares M, Senneville E, IWGDF Editorial Board. Practical guidelines on the prevention and management of diabetes-related foot disease (IWGDF 2023 update). Diabetes Metab Res Rev. 2024;40(3):e3657. 10.1002/dmrr.3657. 37243927 10.1002/dmrr.3657

[43] Chen P, Vilorio NC, Dhatariya K, Jeffcoate W, Lobmann R, McIntosh C, Piaggesi A, Steinberg J, Vas P, Viswanathan V, Wu S, Game F. Guidelines on interventions to enhance healing of foot ulcers in people with diabetes (IWGDF 2023 update). Diabetes Metab Res Rev. 2024;40(3):e3644. 10.1002/dmrr.3644. 37232034 10.1002/dmrr.3644

[44] Senneville É, Albalawi Z, van Asten SA, Abbas ZG, Allison G, Aragón-Sánchez J, Embil JM, Lavery LA, Alhasan M, Oz O, Uçkay I, Urbančič-Rovan V, Xu ZR, Peters EJG. IWGDF/IDSA guidelines on the diagnosis and treatment of diabetes-related foot infections (IWGDF/IDSA 2023). Diabetes Metab Res Rev. 2024;40(3):e3687. 10.1002/dmrr.3687. 37779323 10.1002/dmrr.3687

[45] Dumville JC, Lipsky BA, Hoey C, Cruciani M, Fiscon M, Xia J. Topical antimicrobial agents for treating foot ulcers in people with diabetes. Cochrane Database Syst Rev.2017(6):CD11038. 10.1002/14651858.CD011038.pub2 PMC6481886 28613416

[46] Edmonds M, Lázaro-Martínez JL, Alfayate-García JM, Martini J, Petit JM, Rayman G, et al. Sucrose octasulfate dressing versus control dressing in patients with neuroischaemic diabetic foot ulcers (Explorer): an international, multicentre, double-blind, randomised, controlled trial. Lancet Diabetesendocrinol. 2018;6(3):186–96. 10.1016/S2213-8587(17)30438-2 29275068

[47] Santema TBK, Poyck PPC, Ubbink DT. Systematic review and meta-analysis of skin substitutes in the treatment of diabetic foot ulcers: Highlights of a Cochrane systematic review. WoundRepairRegen. 2016;24(4):737–44. 10.1111/wrr.12434 27062201

[48] Hitchman LH, Totty JP, Raza A, Cai P, Smith GE, Carradice D, et al. Extracorporeal shockwave therapy for diabetic foot ulcers: a systematic review and meta-analysis. Ann Vasc Surg. 2019;56:330–9. 30496896 10.1016/j.avsg.2018.10.013

[49] Wukich DK, Schaper NC, Gooday C, Bal A, Bem R, Chhabra A, Hastings M, Holmes C, Petrova NL, Santini AMG, Senneville E, Raspovic KM. Guidelines on the diagnosis and treatment of active Charcot neuro-osteoarthropathy in persons with diabetes mellitus (IWGDF 2023). Diabetes Metab Res Rev. 2024;40(3):e3646. 10.1002/dmrr.3646. 37218537 10.1002/dmrr.3646

